# An assessment of the impact of a new-type of doctor–nurse joint prenatal breastfeeding clinic attendance on women’s breastfeeding duration and feeding-related breast problems: A cohort observational study

**DOI:** 10.1097/MD.0000000000042984

**Published:** 2025-06-20

**Authors:** Xingxing Liu, Xiangping He, Guihua Xiao, Wei Yuan, Jinhe Xiao

**Affiliations:** a Haidian District Maternal and Child Health Hospital, China.

**Keywords:** breastfeeding duration, breastfeeding problems, doctor–nurse joint, prenatal breastfeeding clinic

## Abstract

To promote breastfeeding, a new-type of doctor–nurse joint prenatal breastfeeding clinic was carried out in Haidian District Maternal and Child Health Hospital, Beijing, China. Actual improvement in the duration of breastfeeding and feeding-related breast problems for women using these clinics remains unknown. This observational study assesses the impact of doctor–nurse joint prenatal breastfeeding clinic’s interventions on breastfeeding duration and feeding-related breast problems in comparison with women who did not receive clinic services. Pregnant women with more than 36 weeks of gestation in Haidian District Maternal and Child Health Hospital were enrolled from July 2023 to September 2023. Patients were grouped based on whether they attended the doctor–nurse joint prenatal breastfeeding clinic. Telephone follow-up calls were conducted at 1 and 6 months postpartum. A total of 90 sets of valid data were collected, including 37 in the target group and 53 in the comparison group. There were no statistically significant differences between the 2 groups with respect to the following metrics: the rate of exclusive breastfeeding at 1 and 6 months postpartum, incidence of nipple injury and mastitis at 1 month postpartum, duration of exclusive and overall breastfeeding, and costs of breast treatment. The main reason for ceasing breastfeeding was milk insufficiency. The modality of lactation suppression was mainly natural cessation. Breast discomforts were mainly milk stasis. There is no significant advantage of doctor–nurse joint prenatal breastfeeding clinic. The promotion of breastfeeding still needs to explore new ways or comprehensive methods.

**Key points**

• In order to promote breastfeeding, a new-type of doctor–nurse joint prenatal breastfeeding clinic was carried out in Haidian District Maternal and Child Health Hospital, Beijing, China.
• Actual improvement in the duration of breastfeeding and feeding-related breast problems for women using the clinic remains unknown.
• This observational study assessed the impact of the clinic’s interventions on breastfeeding duration and feeding-related breast problems among women in comparison with women who did not receive clinic services.
• The study found no significant benefit.

## 1. Background

The period from birth to 2 years of age is a critical phase in shaping the nutritional and health status of children throughout their lifespans.^[1]^ Breastfeeding is the best source of nutrition for infants and young children during this period and is one of the most effective measures to ensure healthy growth and development.^[2]^ Breastfeeding is defined as the child receiving breast milk directly from the breast or expressed milk.^[3]^ World Health Organization, recommends exclusive breastfeeding for the first 6 months of life. After this period, infants should be introduced to solid foods and continue to be breastfed for up to 2 years or more.^[4]^ Improved breastfeeding practices could save 8,20,000 lives each year, 87% of which are infants under 6 months of age.^[5]^ The rate of exclusive breastfeeding in China is 29.2%,^[6]^ which is lower than the global average of 43%.^[1]^ Research shows that breastfeeding education during pregnancy in healthcare facilities plays an important role in helping mothers learn about breastfeeding before delivery.^[6]^ Prenatal breastfeeding education increased breastfeeding uptake postpartum.^[7]^

The traditional breastfeeding clinic is conducted by nurses. Women come to the clinic with actual breastfeeding problems. Nurses provide health education counseling and interventions according to the actual problems the patients encountered while breastfeeding. Nurses may not be able to effectively prevent and intervene breastfeeding problems due to inadequate ability to detect breast and other systemic diseases in the early stage. Based on this, Maternal and Child Health Hospital of Haidian District, Beijing, China, has carried out a doctor–nurse joint prenatal breastfeeding clinic. The study aimed to assess the impact of a new-type of doctor–nurse joint breastfeeding clinic’s interventions on breastfeeding duration and the incidence of feeding-related breast problems in comparison with women who did not receive clinic services.

## 2. Materials and methods

### 2.1. Research design

This is a cohort observational study.

### 2.2. Setting

The study was undertaken at the Maternal and Child Health Hospital of Haidian District, Beijing, China. Beijing is the capital of China, and its metropolitan area is home to about 20 million people. The hospital serves a population that is mostly from Beijing and some patients who live in other areas. More than 10,000 women give birth at the hospital every year. Most of these pregnant women have no breastfeeding experience.

### 2.3. Population and sampling criteria

Inclusion criteria: Pregnant women with more than 36 weeks of gestation in Maternal and Child Health Hospital of Haidian District, Beijing, China, were enrolled from July 2023 to September 2023.

Exclusion criteria: those who are unable to breastfeed due to infectious diseases, mental disorders, or other reasons; those who are not singleton pregnancies; those who gave birth to an infant born with an abnormality or serious illness; those who are unable to cooperate with the study.

All pregnant women were grouped into target group (composed of women having used the services of the breastfeeding clinic) and comparison group (composed of women with no clinic services) based on whether they attended the doctor–nurse joint prenatal breastfeeding clinic.

### 2.4. *Doctor–nurse joint prenatal breastfeeding clinic*

All team members were trained in the core curriculum of internationally certified lactation support providers. For pregnant women attending the doctor–nurse joint prenatal breastfeeding clinic, the doctor conducts a breast physical examination for detection of breast disease, the nurse conducts health education for 10 minutes with reference to the pamphlet “Breastfeeding, the Irreplaceable Love of the Child” edited by the Maternal and Child Health Hospital of Haidian District, Beijing, and provides technical support on the basic skills of breastfeeding on feeding posture and manual breast milk expression for 10 minutes. They will answer questions from pregnant women and give individualized breastfeeding advice. For health problems and needs, multidisciplinary consultation will be provided.

### 2.5. Data collection

Telephone follow-up calls were conducted at 1 month and 6 months postpartum.

The following data were collected from all enrolled pregnant women: demographic information, medical history, parity, sex of the fetus, breastfeeding experience, and whether they attended a joint healthcare breastfeeding clinic.

The phone follow-up was composed of following themes: whether breastfeeding was exclusive, the duration of exclusive and overall breastfeeding within 6 months postpartum, the incidence of feeding-related breast problems (nipple injury and mastitis) at the first month postpartum, the reasons for ceasing breastfeeding, the costs of breast treatment (including medical costs, costs of breast massage by nonmedical staff and overall costs), the modalities of lactation suppression and the frequency of breast discomforts.

### 2.6. Data analysis

The demographic data regarding sex of the fetus, parity, breastfeeding experience, and exclusive breastfeeding at 1 month and 6 months postpartum, nipple laceration and mastitis at 1 month postpartum were expressed in a frequency distribution. Gestational age were expressed as mean (standard deviation). The duration of exclusive and overall breastfeeding (days), the costs of breast treatment (yuan) was non-normally distributed data and was expressed as median (interquartile range).

Chi-square tests were used to search for associations between the variables studied (sex of the fetus, parity, breastfeeding experience, and exclusive breastfeeding at 1 month and 6 months postpartum, nipple injury). Fisher’s exact tests were used to examine the difference in the incidence of mastitis at 1 month postpartum. *T*-tests was used to examine the difference in the maternal age between the 2 groups. Rank-sum test was used to examine the difference in the duration of exclusive and overall breastfeeding (days), the costs of breast treatment (yuan) between the 2 groups. Descriptive analysis was performed on reasons for breastfeeding cessation, breast symptoms and modalities of lactation suppression. The threshold for significance was set at *P* < .05. All the analyses were performed using Statistical Package for the Social Sciences (SPSS) (Version 29.0).

## 3. Results

Table 1 shows the general information and clinical characteristics of the patients.

**Table 1:** General information and clinical characteristics of the patients.

Characteristics	Target group (n = 37) *M* (SD) 或N (%)	Comparison group (n = 54) *M* (SD) 或N (%)	*t*/*χ*^2^	*P*
Maternal age, *y*	31.62 (3.25)	33.50 (4.22)	2.287	.025
Parity			7.079	.029
1	28 (75.7)	29 (54.7)		
2	6 (16.2)	22 (41.5)		
3	3 (8.1)	2 (3.8)		
Sex of the fetus			0.135	.713
Male	21 (56.8)	28 (52.8)		
Female	16 (43.2)	25 (47.2)		
Breastfeeding experience			3.459	.063
No	28 (75.7)	30 (56.6)		
Yes	9 (24.3)	23 (43.4)		

Values are given as mean (SD) or N (%).

Chi-square tests were used to examine the difference in the sex of the fetus, parity and breastfeeding experience between the 2 groups.

*T*-tests was used to examine the difference in the maternal age between the 2 groups.

*P* value of <.05 was considered statistically significant. SD = standard deviation.

From July 2023 to September 2023, a total of 164 pregnant women with more than 36 weeks of gestation were enrolled in the Maternal and Child Health Hospital of Haidian District. Excluding 1 twin pregnancy and 73 could not be reached, a total of 90 participants were left, of which 37 in the target group and 53 in comparison group. Pregnant women in the target group were younger (31.62 vs 33.5, *P* = .025) and had a higher proportion of primiparous women (75.7% vs 54.7%, *P* = .029).

Table 2 shows the information of breastfeeding, feeding-related breast problems and costs of breast treatment.

**Table 2:** Information of breastfeeding, feeding-related breast problems and cost of breast treatment.

Characteristic	Target group (n = 37) N (%) or *M* (IQR)	Comparison group (n = 54) N (%) or *M* (IQR)	*χ*^2^/*Z*	*P*
Exclusive breastfeeding at 1 mo postpartum			1.562	.211
No	12 (32.4)	11 (20.8)		
Yes	25 (67.6)	42 (79.2)		
Nipple injury at 1 mo postpartum			0.562	.453
No	31 (83.8)	41 (77.4)		
Yes	6 (16.2)	12 (22.6)		
Mastitis at 1 mo postpartum				.641
No	36 (97.3)	50 (94.3)		
Yes	1 (2.7)	3 (5.7)		
Exclusive breastfeeding at 6 mo postpartum			0.037	.848
No	16 (43.2)	24 (45.3)		
Yes	21 (56.8)	29 (54.7)		
Duration of exclusive breastfeeding (d)	114.6 (175.5)	117.5 (150)	−0.149	.881
Duration of overall breastfeeding (d)	163.4 (0)	165.8 (90)	−0.670	.503
Total costs (yuan)	943.2 (1350)	825.8 (1200)	−0.429	.668
Costs of breast massage by nonmedical staff (yuan)	648.6 (1200)	669.1 (1000)	−0.058	.954
Medical costs (yuan)	294.6 (0)	164.3 (0)	−0.041	.967

Values are given as N (%) or *M* (IQR).

Rank-sum test was used to examine the difference in the duration of exclusive and overall breastfeeding (d), the cost of breast treatment (yuan) between the 2 groups.

Fisher’s exact tests were used to examine the difference in the mastitis at 1 mo postpartum between the 2 groups.

Chi-square tests were used to examine the difference in the exclusive breastfeeding at 1 mo and 6 mo postpartum, nipple injury between the 2 groups.

*P* value of <.05 was considered statistically significant. IQR = interquartile range.

There were no statistically significant differences (*P* > .05) in the target group compared to the comparison group with respect to the following metrics: the rate of exclusive breastfeeding at 1 month (67.6% vs 79.2%, *P* = .211) and 6 months (56.8% vs 54.7%, *P* = .848) postpartum, the incidence of nipple injury (16.2% vs 22.6%, *P* = .453) and mastitis (2.7% vs 5.7%, *P* = .0641) at 1 month postpartum, the duration of exclusive (114.6 vs 117.5, *P* = .881) and overall breastfeeding (163.4 vs 165.8, *P* = .503), medical costs (294.6 vs 164.3, *P* = .967), costs of breast massage by nonmedical staff (648.6 vs 669.1, *P* = .954), total costs (943.2 vs 825.8, *P* = .668).

Reasons for ceasing breastfeeding were mainly milk insufficiency (6 vs 6), infant refusal (1 vs 1), milk stasis (1 vs 1), work reasons (1 vs 0), milk allergy (0 vs 1), and admission of fetus to the hospital for treatment (0 vs 1). The modality of lactation suppression was mainly natural cessation (6 vs 8), and treatment of traditional Chinese medicine (1 vs 1). Frequency of breast discomforts: milk stasis (61 vs 235), nipple injury (17 vs 79), mastitis (10 vs 17), breast engorgement (5 vs 3), itchy skin sensation (2 vs 0).

## 4. Discussion

The purpose of this study was to assess the impact of a new-type of doctor–nurse joint prenatal breastfeeding clinic attendance on women’s breastfeeding duration and feeding-related breast problems. The results indicate that the clinic has no statistically significant advantage on the rate of exclusive breastfeeding at 1 month and 6 months postpartum, the incidence of nipple injury and mastitis at 1 month postpartum, the duration of exclusive and overall breastfeeding and the costs of breast treatment. Overall, the doctor–nurse joint prenatal breastfeeding clinic did not show a significant advantage, which may be due to the following reasons: Pregnant women had already undergone breast examination and related disease interventions during the preparation for pregnancy. Breast problems was insufficient to affect breastfeeding. The number of cases was relatively small, and geographic limitations were noted. The breastfeeding of the author’s hospital is at a high level (e.g., exclusive breastfeeding rate 55.6% at 6 months postpartum), and it is difficult to improve it only by breastfeeding clinic, which cannot fully reflect the value of doctor–nurse joint breastfeeding clinic. Pregnant women can obtain breastfeeding-related knowledge from other sources such as the Internet. The number of attendance to the clinic was insufficient, with only 1 time.

The results showed that pregnant women attending the clinic were younger and had a high proportion of primiparous women, suggesting that they may have been more eager to receive breastfeeding support due to their lack of breastfeeding experience, although there was no significant difference in breastfeeding experience between the 2 groups. One multiparous woman did not have breastfeeding experience (she had never breastfed), and still did not attend the clinic, suggesting that there is a need for education for multiparous mothers. The main reason for ceasing breastfeeding was milk insufficiency, however, milk stasis is the most common cause of breast discomfort. It is suggested that the breastfeeding clinic should focus on building the confidence of pregnant women to breastfeed, advocating breastfeeding on infant demand, and teaching the correct breastfeeding position.

Factors affecting breastfeeding are multiple, which can be summarized at the individual, institutional and social structure levels,^[8]^ including mothers’ perception of breastfeeding, maternal and child health, family care, support from workplace, public places, etc. There is a need to explore a comprehensive approach to promote breastfeeding from a high level.

The doctor–nurse joint prenatal breastfeeding clinic is a new type of clinic, and there is no evaluation report on its effectiveness. This article is the first to assess the application effect of this clinic. Long-term breastfeeding-related data were collected during the follow-up period for up to 6 months, and relevant conclusions were drawn. Suggestions and directions for improving the future breastfeeding clinic were proposed.

This study only conducted one-time breastfeeding clinic education session before delivery. Further improvement could focus on conducting the education in multiple sessions or multiple times before delivery or combining the education sessions before and after delivery for a more effective improvement of breastfeeding. Further increase of sample size, multicenter and multiregional studies are needed because this study is limited to the author’s hospital and the sample size is small.

In conclusion, this study suggests that the doctor–nurse joint prenatal breastfeeding does not have significant advantage. Despite the results, the courage and pace of innovation should not stop. This paper informs the construction of a new type of breastfeeding clinic and provides directions for better promotion of breastfeeding.

## 5. Limitations

The longest follow-up period was 6 months postpartum, and it is possible that patients who achieved longer benefits may not have been included in the study. There may have been factors of benefit that were not followed up. Factors such as family care, support from workplace, public places were not included in the analysis. Our sample size was relatively small. There are geographical limitations to the study.

## 6. Conclusion

The doctor–nurse joint prenatal breastfeeding clinic did not have a significant effect on the rate of exclusive breastfeeding at 1 month and 6 months postpartum, the incidence of nipple injury and mastitis at 1 month postpartum, the duration of exclusive and overall breastfeeding, and the costs of breast treatment. The promotion of breastfeeding still requires novel approaches and integrated methods.

## Acknowledgments

The authors wish to thank all the doctors and nurses conduct the clinic and their assistance is gratefully acknowledged. We thank all the pregnant women who participated and cooperated with the follow-up. We sincerely appreciate their help.

## Author contributions

**Conceptualization:** Xingxing Liu, Xiangping He, Guihua Xiao, Wei Yuan.

**Data curation:** Xingxing Liu.

**Formal analysis:** Xingxing Liu, Wei Yuan.

**Investigation:** Xingxing Liu, Wei Yuan.

**Methodology:** Xingxing Liu, Xiangping He, Guihua Xiao, Jinhe Xiao.

**Project administration:** Xiangping He.

**Resources:** Xiangping He, Guihua Xiao, Wei Yuan.

**Software:** Xingxing Liu.

**Supervision:** Xingxing Liu.

**Validation:** Xingxing Liu.

**Visualization:** Xingxing Liu.

**Writing** – **original draft:** Xingxing Liu.

**Writing** – **review & editing:** Xingxing Liu.
